# Improving Aquatic Warbler Population Assessments by Accounting for Imperfect Detection

**DOI:** 10.1371/journal.pone.0094406

**Published:** 2014-04-08

**Authors:** Steffen Oppel, Piotr Marczakiewicz, Lars Lachmann, Grzegorz Grzywaczewski

**Affiliations:** 1 RSPB Centre for Conservation Science, Royal Society for the Protection of Birds, The Lodge, Sandy, Bedfordshire, United Kingdom; 2 Ogólnopolskie Towarzystwo Ochrony Ptaków, Marki, Poland; 3 Biebrzański Park Narodowy, Osowiec Twierdza 8, Goniądz, Poland; 4 University of Life Sciences, Department of Zoology, Animal Ecology and Wildlife Management, Lublin, Poland; University of Lleida, Spain

## Abstract

Monitoring programs designed to assess changes in population size over time need to account for imperfect detection and provide estimates of precision around annual abundance estimates. Especially for species dependent on conservation management, robust monitoring is essential to evaluate the effectiveness of management. Many bird species of temperate grasslands depend on specific conservation management to maintain suitable breeding habitat. One such species is the Aquatic Warbler (*Acrocephalus paludicola*), which breeds in open fen mires in Central Europe. Aquatic Warbler populations have so far been assessed using a complete survey that aims to enumerate all singing males over a large area. Because this approach provides no estimate of precision and does not account for observation error, detecting moderate population changes is challenging. From 2011 to 2013 we trialled a new line transect sampling monitoring design in the Biebrza valley, Poland, to estimate abundance of singing male Aquatic Warblers. We surveyed Aquatic Warblers repeatedly along 50 randomly placed 1-km transects, and used binomial mixture models to estimate abundances per transect. The repeated line transect sampling required 150 observer days, and thus less effort than the traditional ‘full count’ approach (175 observer days). Aquatic Warbler abundance was highest at intermediate water levels, and detection probability varied between years and was influenced by vegetation height. A power analysis indicated that our line transect sampling design had a power of 68% to detect a 20% population change over 10 years, whereas raw count data had a 9% power to detect the same trend. Thus, by accounting for imperfect detection we increased the power to detect population changes. We recommend to adopt the repeated line transect sampling approach for monitoring Aquatic Warblers in Poland and in other important breeding areas to monitor changes in population size and the effects of habitat management.

## Introduction

Surveying animal populations to estimate abundance and changes in population size over time is a fundamental goal in ecology and conservation. Very few bird species are so easy to detect and enumerate that accurate estimates of abundance could be obtained without the need to correct for birds that are missed during surveys [1]–[3]. Over the past decades, numerous techniques have been developed to account for the imperfect detection process during bird surveys in order to estimate abundance or density of populations [4]–[13]. For many conservation practitioners, these survey designs and analytical techniques have been either too cumbersome or technically too challenging to implement [14], so that simple indices of abundance are still widely used for many bird monitoring or conservation projects [14]–[16]. However, accounting for imperfect detection is critical even for relative comparisons over time or between experimental units to avoid erroneous conclusions [17]–[19]. Especially for species that depend on conservation management, monitoring changes over time is essential to assess whether management is achieving conservation targets.

Many bird species of temperate grasslands depend either on low-intensity agriculture or specific conservation management to maintain suitable breeding habitat. One such species is the Aquatic Warbler (*Acrocephalus paludicola*), a small passerine bird species that breeds in broad lowland river valleys, mainly on mesotrophic and slightly eutrophic sedge fen mires in central Europe [20]–[22]. The species is globally threatened (‘Vulnerable’), and breeding habitats are in danger of being lost due to agricultural land abandonment and eutrophication. The Aquatic Warbler is therefore dependent on ongoing landscape-scale management that limits natural succession and prevents breeding habitat from overgrowing [23]–[25]. Because the species is a long-distance migrant that winters in sub-Saharan Africa [26]–[28], processes outside the breeding season may also affect the population size of the species [29]. Robust monitoring of Aquatic Warbler breeding populations is therefore necessary to assess whether habitat management on breeding grounds is sufficient and effective in maintaining stable breeding populations.

Aquatic Warbler populations in Central Europe have traditionally been surveyed with a chain of observers spaced at intervals that are believed to be small enough to facilitate detection of every singing male Aquatic Warbler [30]. Numbers obtained from those surveys have been routinely added up over many sites in order to calculate population sizes of Aquatic Warblers [23]. These traditional survey techniques have been retained to ensure consistency in counting methodology over many years and to provide distribution data for land managers. However, this approach provides no estimate of precision and does not account for observation error. Differences in detection probability may occur in different habitats, and may not remain constant over time [17], [19], [31]–[32]. The issue of imperfect detection is especially important for Aquatic Warbler surveys due to the dynamic habitat conditions with continuously changing vegetation height between years. Simple counts that assume that detection probability is constant may therefore hamper the ability to detect population trends or assess the effects of habitat management for Aquatic Warblers.

Two common bird monitoring approaches that address the problem of imperfect detection are distance sampling and repeated surveys using multiple visits or multiple observers [1]. Distance sampling accounts for the generally declining probability of detecting a bird with increasing distance from the observer [13]–[14], [33], but assumes that distances to detected birds can be measured accurately. In Aquatic Warblers most birds are only recorded acoustically in a structurally uniform environment, and distance estimation is challenging and potentially unreliable [34]–[35]. By contrast, repeated surveys are technically easy to implement, and recent analytical developments (binomial mixture models) allow the estimation of detection probability from repeated counts [6], [11], [36]–[37]. These binomial mixture models provide an excellent basis for analysing population trends over time while accounting for imperfect detection [9].

We established a repeated line transect survey monitoring scheme at one of the largest contiguous breeding habitats of Aquatic Warblers, the Biebrza valley in eastern Poland, to evaluate whether this monitoring programme can achieve monitoring targets and provide information to assess the efficacy of landscape management for the conservation of Aquatic Warblers. We first describe the survey design and the approach used to estimate abundance of Aquatic Warblers from repeated line transect surveys in 2011 – 2013. We then conducted a power analysis to assess whether the line transect monitoring approaches could meet the objectives specified in the Aquatic Warbler species action plan [23], [38]. This analysis thus provides an assessment of both the accuracy and practical feasibility of adopting a new survey design for the only globally threatened songbird of mainland Europe.

## Methods

### Ethics statement

All fieldwork was authorised by the Biebrzański Park Narodowy authority. No animals were captured or handled, and no harm was inflicted on any wild animal population.

### Study area and transect design

**Figure 1:**
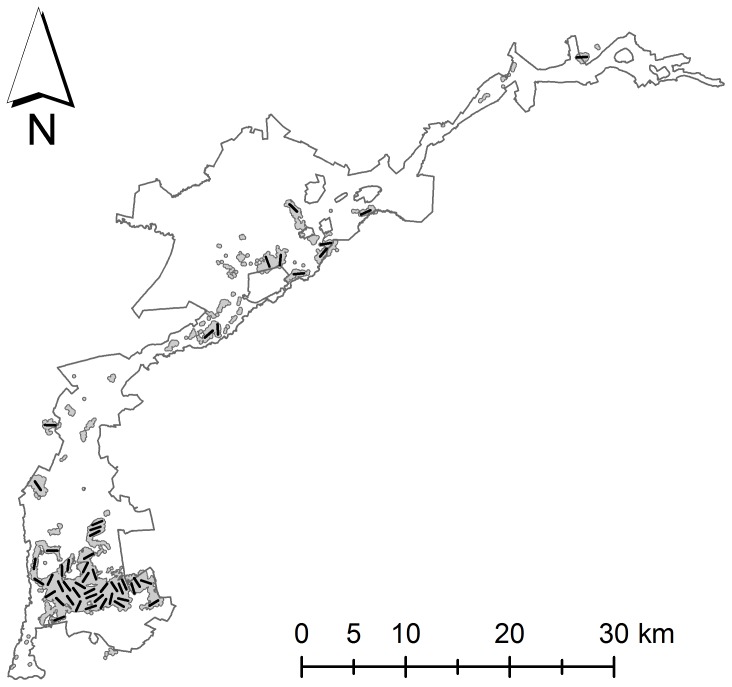
Outline of the study area in the Biebrza valley, Poland, indicating the location of 50 1-km transects along which singing male Aquatic Warblers were surveyed in 2011 and 2012. The outline represents the border of the Biebrza National Park, the grey shaded areas depict suitable Aquatic Warbler habitat that is surveyed during the ‘full count’ approach.

The Biebrza valley, Poland (53°16′N, 22°33′E) holds one of the largest populations of Aquatic Warblers in the world [23]. Since 2003, population surveys have relied on a large number of volunteers that attempted to count every singing male by surveying the 8280 ha of suitable habitat in and adjacent to the Biebrza National Park over 35 consecutive days during the breeding season between May and August. In 2011, we placed 50 1-km transects within suitable Aquatic Warbler nesting habitat (Fig. 1). Transects were placed using a random starting point and a random direction, and constrained to lie within the irregularly shaped 8280 ha of suitable habitat and at least 500 m apart from the nearest transect. Because habitat management in the Biebrza National Park is ongoing and patchily distributed, transects could contain habitat ranging from recently mown very short vegetation (< 40 cm) to taller sedges (> 120 cm) mixed with reeds and bushes up to 5 m in height. Visual inspection of transects prior to the breeding season confirmed that vegetation composition on transects was representative of vegetation composition of suitable Aquatic Warbler habitat in the Biebrza valley.

### Bird surveys

In May and June 2011 – 2013, Aquatic Warblers were surveyed on calm evenings from 1 hr before to 1 hr after sunset, which is the time of maximum singing activity that increases detection of singing males [30]. One observer walked the centreline of each transect in 30–60 minutes and recorded all singing males. Observers kept track of locations of singing males on a map to avoid repeatedly counting the same individual.

For each transect, three replicate surveys were conducted within one week between 20 May and 25 June in all years. This period corresponds to the first of two breeding peaks of the species [39]–[40]. Habitat characteristics that are important for Aquatic Warbler distribution [41], namely water depth, vegetation height, and the thickness of the litter layer, were visually estimated at five equally spaced points along each transect (every 200 m). Vegetation height was recorded in four categories: <40 cm, 41–80 cm, 81–120 cm, and >120 cm; litter cover was recorded in four categories: <10%, 10–50%, 50–100%, 100% and >15 cm thick; and water height was also recorded in four categories: dry, wet after trampling, < 15 cm, > 15 cm above ground. For analysis, we used the proportion of each transect that was in a given category, thus resulting in four variables each for vegetation height, water depth, and litter cover. Daily weather data (temperature, precipitation, and wind speed) associated with the survey dates were retrieved from the Bialystok weather station (< 50 km from the study area) via an online data portal (www.weatherunderground.com).

Besides the 50 transects surveyed for this study, 8280 ha of the Biebrza valley were surveyed by several volunteers using the traditional ‘full count’ approach in 2012. For the ‘full count’ surveys, observers walked in parallel c. 70 m apart and recorded every singing male Aquatic Warbler on a map, after comparing notes from adjacent observers to avoid double-counting. On each evening a survey block ranging in size from 50 – 250 ha was covered, and the number of singing males recorded on all survey blocks was added up to obtain one ‘population estimate’ for 2012 (following the general guidelines in [23]).

### Binomial mixture modelling approach

We used the transect survey data to estimate Aquatic Warbler abundance with binomial mixture models [6], [11], [37]. These models use the repeated observations on a given transect to separately estimate the probability to detect birds and the number of birds that use the habitat on and around the transect. Briefly, these models consist of two components which link the ecological state of interest (abundance of birds) and the observation process (detection probability) in a hierarchical fashion. The abundance component is modelled as a random Poisson process and estimates the size of the ‘super-population’ of birds, conceptually the total number of birds whose home range overlaps with the transect area that is covered by observers [6], [11], [18]. The observation model component is conditional on the number of birds estimated on each transect, and estimates the probability of detection based on repeated counts at a given site using binomial trials for each bird. A critical assumption for these models is that the population is closed over the period during which the repeat surveys are conducted. Because Aquatic Warblers can be highly mobile during the breeding season [42]–[43], we conducted repeat surveys of the same transect within one week to satisfy the closure assumption.

The abundance of Aquatic Warblers is dependent on various habitat factors such as vegetation height, litter depth and water depth [21], [25], [41], [44]. To determine which of the habitat variables we measured during transect surveys best accounted for abundance variation between transects, we first fit binomial mixture models with different plausible detection and abundance covariates with the function ‘pcount’ in R package ‘unmarked’ [45] in R 2.15.2 [46]. We considered that abundance might vary with vegetation height, litter cover, or water depth, and that the probability of detecting singing male Aquatic Warblers may be either constant, or depend on mean daily temperature, total daily precipitation, mean daily wind speed, the day of the breeding season (date), or vegetation height. Because the current monitoring assumes that detection probability is similar among years, we specifically tested this assumption by constructing models both with and without year as a categorical detection variable. We did not include time of day, a variable that influences detection probability of many songbirds [19], because the time of day was held constant by the survey design. All models included a temporal trend effect on abundance - the main goal of the annual monitoring. We constructed a total of 45 different candidate models accounting for plausible scenarios of density and detection processes, and we used an information-theoretic approach to select the most parsimonious model based on AIC [47].

After identifying the most important variables influencing detection and abundance, we implemented a final binomial mixture model in a Bayesian framework to estimate abundances per year and population trend following the approach of Kéry et al. [9]. This model differed from the models fitted with the ‘pcount’ function described above in that it recognized that annual surveys of identical transects were not independent by incorporating a random transect effect. We fitted this final model in JAGS 3.3 [48] via the R2jags package [49] called from R 2.15.3 [46]. We used uninformative priors for all parameters and ran three Markov chains each with 350,000 iterations and discarded the first 50,000 iterations as burn-in. To assess whether the model fit the data, we applied a Bayesian posterior predictive check [50], and we report the Bayesian *p*-value as an indicator of model fit [18]. We report posterior mean estimates for abundance, trend and detection probability from this final binomial mixture model (Appendix S1).

### Power analysis to assess whether monitoring can detect target trend

The Aquatic Warbler species action plan aspires to detect a 20% change in the population of Aquatic Warblers over 10 years [23]. To assess whether the revised monitoring design with 50 transects could achieve this target, we conducted a power analysis following the approach by Reynolds et al. [38]. We simulated 1000 population trajectories with a 20% decrease over 10 years, and simulated three transect surveys on each of 50 transects for each year using the random site effects and detection probabilities estimated from our final binomial mixture model. Vegetation height and water height were randomly set for each transect in each year, and abundance and detection probability for each transect were simulated using the water level and vegetation height parameters from our final model (Appendix 1). We then used our final model to estimate population trend for each of the 1000 simulations and estimated the power of this model to detect the target trend as the proportion of simulations where the 95% credible interval of the trend parameter estimate included the actual value (-0.02). To compare whether the transect monitoring in combination with the binomial mixture model provides an improvement over existing survey methods, we used the raw count data summed across the 50 transects to fit a linear regression for each of the 1000 simulations to assess whether the raw count data would be able to detect a trend. We consider the proportion of simulations that detected a significant (*p*<0.05) trend as the power of the traditional monitoring to detect the target population trend.

## Results

The traditional survey was conducted by up to six observers per census area and took 35 days to complete, thus resulting in a total effort of 175 observer days. Repeated transect sampling surveys of 50 transects required one observer per transect survey, and thus a total of 150 observer days per year.

The traditional full count survey in 2012 detected 2594 singing male Aquatic Warblers. The transect survey observations include three repeated visits to each transect, and the total number of singing males during the first, second, and third survey round was 635, 649, and 609, respectively, in 2011 (542, 574, and 608 in 2012, and 622, 551, and 568 in 2013). On average, 12 Aquatic Warblers were detected during each transect survey (SD  =  8.8, range 0 – 51).

**Figure 2:**
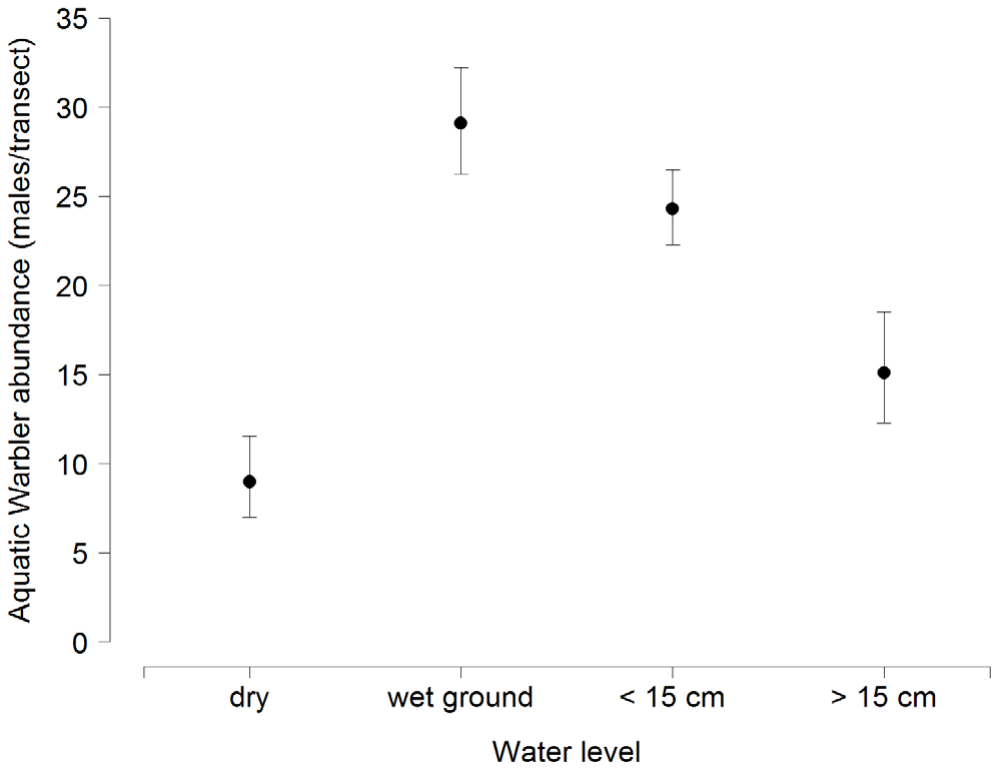
Estimated abundance of singing Aquatic Warbler males on 1-km transects at different water levels in the Biebrza valley, Poland, based on a binomial mixture model with survey data from 2011 – 2013. Error bars represent 95% confidence intervals. Water level was recorded in four categories: dry, wet after trampling, standing water<15 cm above ground, standing water > 15 cm above ground.

**Figure 3:**
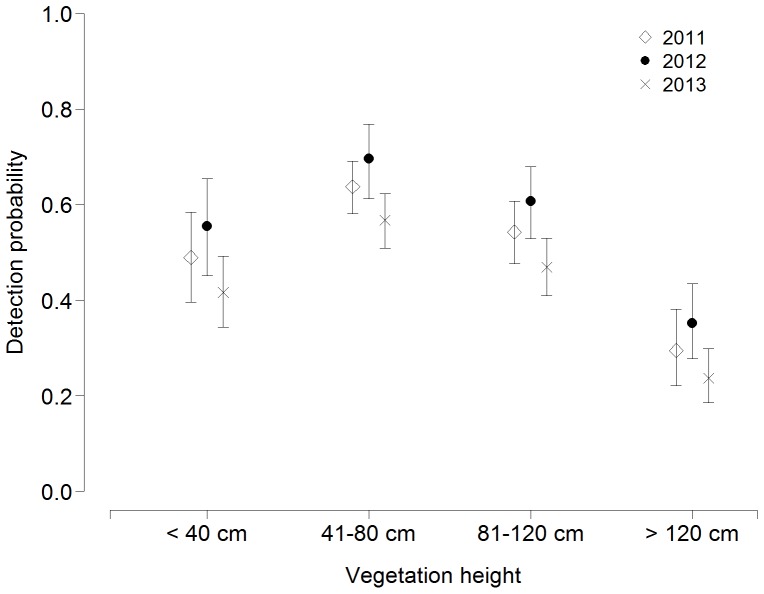
Detection probability of Aquatic Warbler males by a single observer along a 1-km transect at different vegetation heights in different years in the Biebrza valley, Poland, estimated with a binomial mixture model. Error bars represent 95% confidence intervals.

The binomial mixture models that assumed that abundance varied with water level and detection varied by year and with vegetation height received overwhelming support from the data (cumulative AIC weight  =  1.00, Table 1). The most parsimonious model indicated highest Aquatic Warblers abundance at intermediate water levels, and lowest abundance on transects with dry ground (Fig. 2). In addition, the model suggested that detection probability varied between years (Fig. 3) and depended on the amount of rainfall during the day (Table 1).

**Table 1:** Model selection table showing binomial mixture models of Aquatic Warbler abundance in Biebrza valley, Poland, from 2011 to 2013.

Model component variables		*k*	AIC	ΔAIC	ωAIC
Abundance	Detection				
water depth	Year + rain + vegetation height	12	3430.11	0.00	0.88
water depth	Year + vegetation height	11	3435.80	5.69	0.05
water depth	Year + date + vegetation height	12	3436.98	6.87	0.03
water depth	Year + wind + vegetation height	12	3437.76	7.65	0.02
water depth	Year + temperature + vegetation height	12	3437.78	7.67	0.02
water depth	date + vegetation height	10	3449.84	19.73	0.00
water depth	rain + vegetation height	10	3454.70	24.59	0.00
litter cover	Year + rain + vegetation height	12	3454.94	24.83	0.00
water depth	temperature + vegetation height	10	3457.64	27.54	0.00
water depth	vegetation height	9	3457.90	27.79	0.00
litter cover	Year + vegetation height	11	3457.93	27.82	0.00
litter cover	Year + wind + vegetation height	12	3459.77	29.66	0.00
water depth	wind + vegetation height	10	3459.80	29.69	0.00
litter cover	Year + date + vegetation height	12	3459.84	29.73	0.00
litter cover	Year + temperature + vegetation height	12	3459.92	29.81	0.00
litter cover	date + vegetation height	10	3474.87	44.76	0.00
water depth	rain	9	3475.01	44.90	0.00
litter cover	rain + vegetation height	10	3475.40	45.29	0.00
water depth	constant	8	3476.83	46.72	0.00
litter cover	vegetation height	9	3476.85	46.74	0.00

Models have two components accounting for variation in abundance and detection probability, and all models included a temporal trend parameter on abundance. *k*  =  number of estimable parameters, AIC  =  Akaike's information criterion, ΔAIC  =  difference in AIC units to the most parsimonious model, ωAIC  =  relative weight of evidence for each model. Only the best 20 of a total of 45 fitted models are shown, remaining models were not supported by the available data (ΔAIC > 45).

Based on the model selection, we fit a trend model in a Bayesian framework incorporating the effect of water level on abundance and the effects of rain and vegetation height on detection. This model fit the data well (Bayesian *P*-value  =  0.51, slope  =  0.998), and indicated that the estimated abundance of Aquatic Warblers increased by 8% (1 – 17%) between 2011 and 2013 (Table 2), despite similar raw count data.

**Table 2:** Parameter estimates (mean, standard deviation, lower and upper 95% credible intervals) of the most parsimonious binomial mixture model to estimate abundance of singing male Aquatic Warblers in the Biebrza valley, Poland, in 2011 – 2013.

	mean	sd	lcl	ucl
*Abundance parameters*				
wet ground	0.51	0.21	0.10	0.93
standing water <15 cm	0.36	0.20	−0.04	0.76
standing water >15 cm	0.26	0.27	−0.26	0.79
*Detection parameters*				
Rain	0.01	0.06	−0.11	0.13
vegetation height 41 – 80 cm	0.65	0.42	−0.16	1.48
vegetation height 81 – 120 cm	0.70	0.41	−0.10	1.52
vegetation height >120 cm	0.15	0.51	−0.85	1.15
*Derived parameters*				
N Aquatic Warblers (2011)	885	30	837	952
N Aquatic Warblers (2012)	780	46	714	888
N Aquatic Warblers (2013)	1001	65	905	1152
population trend	0.08	0.04	0.01	0.17

The total abundance estimated by the model was 1.36 (95% CrI: 1.29–1.47) times higher than the maximum raw count in 2011, 1.28 (1.17–1.46) times higher than the maximum raw count in 2012, and 1.61 (1.45 – 1.85) times higher than the maximum raw count in 2013. Applying this correction factor to the full count of 2594 Aquatic Warblers in 2012 would result in a population of 3327 (3046 – 3788) singing male Aquatic Warblers in the entire Biebrza valley.

In our power analysis, 68% of 1000 simulations using the repeated surveys and a binomial mixture model were able to detect a 20% change in abundance over 10 years, whereas only 9% of simulations using the raw count data (not correcting for imperfect detection) were able to detect a significant trend.

## Discussion

Accurate and reliable monitoring of breeding populations is critical to detect population declines, which are common for many long-distance migrants in Europe [51]–[53]. Using a randomised line transect survey design with repeated visits and binomial mixture models enabled us to estimate annual Aquatic Warbler abundances, and resulted in much improved power to detect population changes over time. Our power analysis suggested that the revised monitoring had a probability of 68% to detect a 20% decline over 10 years, whereas the power to detect this trend using the raw count data was very low (<10%). This difference is likely a consequence of accounting for imperfect detection: our data indicated that detection probability varied among years (Fig. 3), and any analysis of raw count data that assumes that detection probability is constant between years is therefore less powerful because changes in abundance and changes in detection probability are confounded.

Our analysis of transect surveys between 2011 – 2013 also indicated a moderate increase of the Aquatic Warbler population in the Biebrza valley over these 3 years - a result that was not evident from raw count data. Dedicated habitat management and conservation efforts to improve Aquatic Warbler habitat have been carried out in the Biebrza valley since 2008 [39], [54]–[55], and the benefit of these measures would not have been apparent with the traditional monitoring approach. We therefore conclude that the repeated line transect sampling introduced here will improve assessments of the efficacy of conservation management and will help to meet the targets of the species action plan for the Aquatic Warbler.

Comparing estimated abundance and raw count data indicated that the estimated population size on transects was 1.28 – 1.61 times higher than the raw count data suggested, a result that is consistent with imperfect detection described in many studies [11], [31], [56]. Imperfect detection in Aquatic Warblers may not only be a result of observers failing to detect a singing bird, but may also occur because birds may not be singing on a given day or may be temporarily elsewhere [30], [42]. Such a lack of availability for detection is difficult to control with more observers or close observer spacing, and raw counts will therefore always contain observation errors that may mask long-term population trends [18]. However, the detection probability and abundance estimated for our survey transects cannot necessarily be scaled up to the entire Biebrza valley, and our ‘corrected’ estimate of the Biebrza population size (3327 instead of 2594 singing male Aquatic Warblers) must be interpreted with caution. This complication arises due to the definition of ‘abundance’ estimated by the binomial mixture models for each transect: these models estimate the abundance of a ‘super-population’ of birds that potentially use a transect but may have activity centres outside the transect area. For territorial species, this ‘super-population’ can be interpreted as the number of birds whose territory overlaps with the survey area [5], [18]–[19], but because Aquatic Warbler males are not territorial and may roam considerable distances on breeding grounds [42]–[43], the spatial extent of the ‘super-population’ is very challenging to define in Aquatic Warblers. The high mobility may lead to some individual males being detected at > 1 transect regardless of transect spacing, and may thus lead to an inflated abundance estimate if abundances from transect ‘super-populations’ are extrapolated across the Biebrza valley. The traditional ‘full count’ approach is similarly vulnerable to the mobility of males, because individuals may be counted multiple times on different days. However, males may evade detection during the traditional ‘full count’, while such temporary emigration would be accounted for using the binomial mixture modelling approach. Although the male roaming behaviour may complicate the estimation of the total Biebrza valley population size, abundance estimates of transect ‘super-populations’ would still provide a more robust tool for long-term monitoring than uncorrected raw counts.

All surveys of Aquatic Warblers focus on singing males as the unit of measurement, because females are very difficult to detect owing to their cryptic behaviour. Due to the peculiar breeding system of Aquatic Warblers, it is not clear whether the adult sex ratio is 1∶1, or whether the male population may follow a different trajectory over time than the female population [42]. Estimating the abundance of cryptic population segments is therefore important for conservation, but may require more intensive methods (e.g. mark-recapture) when visual surveys are likely unreliable [2]–[3].

The ability to estimate total population size and the power to detect a moderate trend over time might increase if additional data were available to estimate detection probabilities and actual densities of Aquatic Warblers. Observers at Biebrza record all singing Aquatic Warblers in distance categories, and an earlier analysis using hierarchical distance sampling [5] provided density estimates and revealed increased power to detect the target trend (86%, Oppel, unpubl. data). However, this previous analysis indicated profound differences in the detection probability between observers, which may have been partly due to different accuracies in distance estimation by different observers [35]. We therefore discarded the distance information for the present analysis, but future surveys by highly trained observers with accurate distance estimation abilities may further improve estimates of Aquatic Warbler abundance and population trend.

Besides an improved power to detect population changes over time, the line transect sampling approach introduced here required less effort and fewer observers than the traditional ‘full count’ methodology. However, the observers need to be better qualified to independently conduct transect surveys. Because the availability of highly qualified observers may be a limiting factor in some Aquatic Warbler breeding countries, we caution managers to consider the cost of additional observer training before implementing an altered monitoring design.

In summary, we recommend future monitoring to conduct three replicate surveys along randomly located line transects. This approach is feasible in the field, and facilitates the use of binomial mixture models to estimate annual abundance of Aquatic Warblers with a higher power to detect long-term trends than the traditional monitoring approach.

## Supporting Information

Appendix S1 **Data, R and JAGS code to simulate Aquatic Warbler survey data over 10 years and fit a binomial mixture model to estimate trend for simulated data set.**
(ZIP)
